# Examining the effects of psychological resilience and wellbeing on perceived stress and depressive symptoms among undergraduate nursing interns: testing a moderated mediation model

**DOI:** 10.3389/fpubh.2024.1497076

**Published:** 2024-12-24

**Authors:** Miaomiao Yan, Lijuan Zhang, Dan Qin, Zhongtao Zhou, Yigao Wu, Nuoyu Hou, Xiubin Tao

**Affiliations:** ^1^Nursing Department, The First Affiliated Hospital of Wannan Medical College (Yijishan Hospital of Wannan Medical College), Wuhu, Anhui, China; ^2^College of Nursing, Wannan Medical College, Wuhu, Anhui, China; ^3^Department of General Surgery, The First Affiliated Hospital of Anhui Medical University, Hefei, China; ^4^Anhui Public Health Clinical Center, Hefei, China; ^5^College of Nursing, Bengbu Medical University, Bengbu, Anhui, China; ^6^Department of Psychology, The First Affiliated Hospital of Wannan (Yijishan Hospital of Wannan Medical College), Wuhu, Anhui, China

**Keywords:** perceived stress, depression, psychological resilience, wellbeing, moderated mediation model

## Abstract

**Background:**

Perceived stress is recognized as a significant risk factor for depressive symptoms, while psychological resilience and wellbeing are considered crucial protective factors. However, the intricate relationships among these variables in undergraduate nursing interns remain largely unexplored. This study aims to investigate the mediating role of psychological resilience in the relationship between perceived stress and depressive symptoms, as well as the moderating influence of wellbeing on this mediation.

**Methods:**

From March 1 to 31, 2024, a cluster sampling survey was conducted to examine senior nursing undergraduates from a medical college in Anhui Province who were engaged in clinical practice at various hospitals. The surveyed hospitals were all Grade III, and Class A teaching institutions situated in Anhui, Jiangsu, Zhejiang, Shanghai, and other provinces. According to the Lewinsohn behavioral theory of depression, the study measured perceived stress, depressive symptoms, wellbeing, and psychological resilience using the Chinese Perceived Stress Scale (CPSS), Patient Health Questionnaire 9 (PHQ-9), Oxford Happiness Questionnaire (OHQ), and the Chinese version of the 10-item Connor-Davidson Resilience Scale (CD-RISC-10). The PROCESS v4.0 macro was utilized to evaluate the mediating role of psychological resilience and the moderating role of wellbeing.

**Results:**

A total of 299 valid samples were included. Perceived stress, depressive symptoms, psychological resilience, and wellbeing were significantly correlated. Psychological resilience partially mediated the relationship between perceived stress and depressive symptoms [indirect effect = 0.111, 95% Boot CI (0.065, 0.162)]. Wellbeing also moderated the relationship between psychological resilience and depressive symptoms [B = 0.007, 95% Boot CI (0.003, 0.011)].

**Conclusion:**

The mental health of undergraduate nursing interns warrants attention. To reduce perceived stress and depressive symptoms, interventions should focus on enhancing psychological resilience and wellbeing among these interns.

## 1 Introduction

Depression constitutes a substantial contributor to the global disease burden, impacting over 300 million individuals across all communities worldwide. Approximately one in five individuals experience a depressive episode during their lifetime, making it the primary cause of disability on a global scale (1). The global prevalence of elevated self-reported depressive symptoms from 2001 to 2020 was 34% (2). Researchers at the National Center for Health Statistics estimated that the national prevalence rate of depression in the United States ranges from 5 to 11.5% (3). Approximately 20% of undergraduate nursing students nationwide screened positive for probable depression (4). Several cross-sectional studies have reported that the prevalence of depressive symptoms among Chinese college students ranges from 12.2 to 28.9% (5–7). The estimated prevalence of depression or depressive symptoms among medical students is 27.2% (8). Undergraduate nursing practice students constituted a notable demographic affected by depression (9).

Nursing students must complete 8 months of clinical nursing training at teaching and general medical institutions and hold a relevant academic history certificate to qualify for the Nursing Practitioner Qualification examination. Owing to numerous shifts in the work environment and interpersonal dynamics, the clinical practice phase has evolved into the most demanding stage of nursing education. Theoretical literature has previously underscored clinical practice as a prominent stressor among nursing students (10, 11). Common stressors during clinical training included assignments, workload, and patient care responsibilities. Academic stressors encompassed insufficient leisure time, low grades, examinations, and academic workload (12). During the concluding phase of their practical training, nursing students experienced diminished initiative and productivity, requiring them not only to fulfill their initial practicum obligations but also to engage in vocational certification, employment assessments, and other examinations, thereby experiencing heightened negative emotions stemming from compounded stress factors (13). Perceived stress refers to the psychological response of individuals to environmental stimuli following cognitive appraisal (14). Research has demonstrated that perceived stress can elevate the risk of developing depression (15, 16). Lewinsohn's behavioral theory of depression posited stress-induced depression by diminishing positive reinforcement stimuli (17). This study is grounded in Lewinsohn's theory, suggesting that increasing positive reinforcement, such as psychological resilience and wellbeing, can reduce stress-induced depression.

According to the resilience theories (18), psychological resilience is a dynamic process of positive adaptation to stress or adversity, characterized by an individual's ability to return to a positive state following significant stressors or adverse conditions, and it can serve as a mediating factor in facilitating personal growth in the context of adversity (19, 20). Psychological resilience can mitigate the incidence of depression among medical students (21). Wellbeing serves as a crucial indicator of both physical and mental health. The greater the perceived stress an individual experiences, the stronger the feelings of unwellbeing and the higher the susceptibility to depression (22). Low levels of wellbeing among university students have been reported globally and have garnered significant attention (23).

Perceived stress, psychological resilience, and wellbeing each play important roles in depressive symptoms, yet their specific influences on these symptoms among undergraduate nursing interns remain unclear. This study aims to explore whether psychological resilience mediates the relationship between perceived stress and depressive symptoms and to evaluate a moderated mediation model. It is hypothesized that psychological resilience may serve as a mediator in the relationship between perceived stress and depressive symptoms among interns. Additionally, wellbeing might play a role as a moderator in the direct and/or indirect effect (including path a: perceived stress → psychological resilience; path b: psychological resilience → depressive symptoms) of perceived stress on depressive symptoms, respectively.

## 2 Methods

### 2.1 Participants and procedures

#### 2.1.1 Participants

From March 1 to 31, 2024, a cluster sampling survey was conducted to examine senior nursing undergraduates from a medical college in Anhui Province who were engaged in clinical practice at various hospitals. The surveyed hospitals were all Grade III, and Class A teaching institutions situated in Anhui, Jiangsu, Zhejiang, Shanghai, and other provinces.

Inclusion criteria: ①At least 18 years old; ②An internship duration exceeding 10 months. ③Voluntary participation with informed consent. Exclusion criteria included: ①Having a mental illness; ②Being diagnosed with a mental disorder or currently taking psychiatric medication.

#### 2.1.2 Sample size calculation

According to Fritz and MacKinnon's simulations of mediation models, a sample size of 71 is necessary to achieve a power of 0.80 for a mediation model evaluated using bias-corrected bootstrapping with medium effect sizes (*d* = 0.39) for the a and b paths (24). Similarly, a priori power analyses conducted using G^*^Power indicate that a sample size of 85 is adequate for the most complex analyses performed (24, 25).

The sample size was determined using G^*^Power 3.1.9.7. A *post-hoc* analysis using G^*^Power was conducted to calculate the achieved power (1 – β) for the sample size of the study. Given that the PROCESS macro utilized a multiple regression model, a fixed linear multiple regression model was employed as the statistical test. The parameters used in the calculations were as follows: an effect size *f*^2^ of 0.15, a significance level α of 0.05, and a total of 5 predictors. The minimum required sample size was determined to be 92. A total of 328 undergraduate nursing interns were recruited to participate in this study. Of the 328 collected questionnaires, 299 were deemed valid, resulting in an effective response rate of 91.16%. A total of 29 questionnaires were manually excluded due to identical responses across all items, indicating a lack of attention during the completion of the questionnaire. The analysis revealed that the regression model achieved a statistical power of 100%, confirming that the sample size of 299 undergraduate nursing interns was sufficient.

#### 2.1.3 Quality control

Internal consistency coefficients were meticulously calculated for all scales employed in the present study. The questionnaire could be completed anonymously online survey. Basic questions were included in the questionnaire to ensure that all submitted responses met the inclusion and exclusion criteria for the sample. Failure to answer questions resulted in the submission being deemed invalid. During data preprocessing, Responses with clearly inconsistent answers (e.g., where all answers were identical, suggesting careless completion of the questionnaire) was manually excluded.

#### 2.1.4 Ethical considerations

The study was conducted by the Declaration of Helsinki, and approved by the Ethics Committee of Yijishan Hospital of Wannan Medical College. All participants were given informed consent before completing the measures. All information remained confidential and anonymous.

### 2.2 Variables and measures

#### 2.2.1 Sociodemographic questionnaire

Data were collected using custom-designed questionnaires that included questions on age, sex, family location, family type, single-child status, and annual household income. All data points were self-reported.

#### 2.2.2 Measurement of perceived stress

The Perceived Stress Scale-14 (PSS-14) was initially developed by Cohen et al. (14) and subsequently revised by Yang and Huang (26). The Chinese Perceived Stress Scale (CPSS-14) comprises two dimensions: a sense of loss of control and a sense of tension, encompassing a total of 14 questions. A 5-point Likert scale was employed, with seven items (4, 5, 6, 7, 9, 10, 13) scored in reverse. The total score ranged from 0 to 56, with higher scores indicating greater perceived stress. The Cronbach's α coefficient of the scale in this study was 0.767.

#### 2.2.3 Measurement of depressive symptoms

Depressive symptoms were assessed using the Patient Health Questionnaire-9 (PHQ-9), which had been used as a screening tool for depression in college students (27).

The PHQ-9, developed in 1999, originates from the Primary Care Evaluation of Mental Disorders (PRIME-MD). It serves as a pivotal depression screening tool in primary care settings. Translated into over 80 languages, the PHQ-9 enjoys extensive global utilization (28). The PHQ-9 exhibits strong reliability and validity in both the Chinese general population and among college students (28, 29). The PHQ-9 comprises nine items, each rated on a 4-point scale ranging from 0 to 3. The total score can range from 0 to 27, with severity levels classified as follows: normal (0–4 points), mild depression (5–9 points), moderate depression (10–14 points), moderately severe depression (15–19 points), and severe depression (20–27 points). A cutoff score of 10 or above is typically regarded as indicative of a current depressive episode, aligning with classifications of moderate to severe depression (30). The Cronbach's α coefficient of the scale in this study was 0.851.

#### 2.2.4 Measurement of psychological resilience

Campbell-Sills and Stein (31) investigated the psychometric properties of the 10-item Connor-Davidson Resilience Scale (CD-RISC-10). Ye et al. (32) introduced the scale to China and conducted localization, as well as reliability and validity tests, resulting in the creation of a Chinese version of the CD-RISC-10 scale. Participants responded using a 5-point Likert scale, with total scores ranging from 0 to 40; higher scores signifying higher levels of psychological resilience. The Cronbach's α coefficient of the scale in this study was 0.843.

#### 2.2.5 Measurement of wellbeing

The Oxford Happiness Questionnaire (OHQ), comprising 29 items, was utilized to collect data about wellbeing. A 6-point Likert scale was employed (1 = strongly disagree, 6 = strongly agree). Of the 29 items, 12 were reverse-scored. The total scores ranged from 6 to 174. The final score, ranging from 1 to 6, was obtained by dividing the total sum by 29. The final score categories were as follows: 1–2: “not happy,” 2–3: “somewhat unhappy,” 3–4: “neither happy nor unhappy,” 4: “somewhat happy,” 4–5: “rather happy/pretty happy,” 5–6: “very happy,” and 6: “extremely happy.” Higher scores indicate greater levels of wellbeing (33). The Cronbach's α coefficient of the scale in this study was 0.894.

### 2.3 Statistical analysis

**Figure 1:**
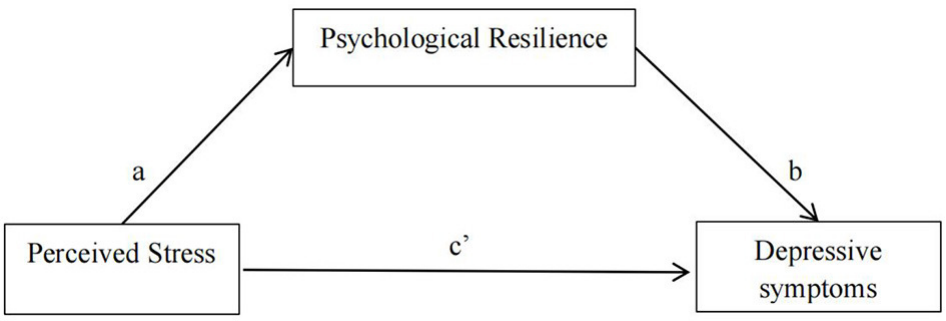
A schematic representation of psychological resilience acting as a mediator between perceived stress and depressive symptoms (Andrew Hayes's Moderation-Mediation Model 4).

**Figure 2:**
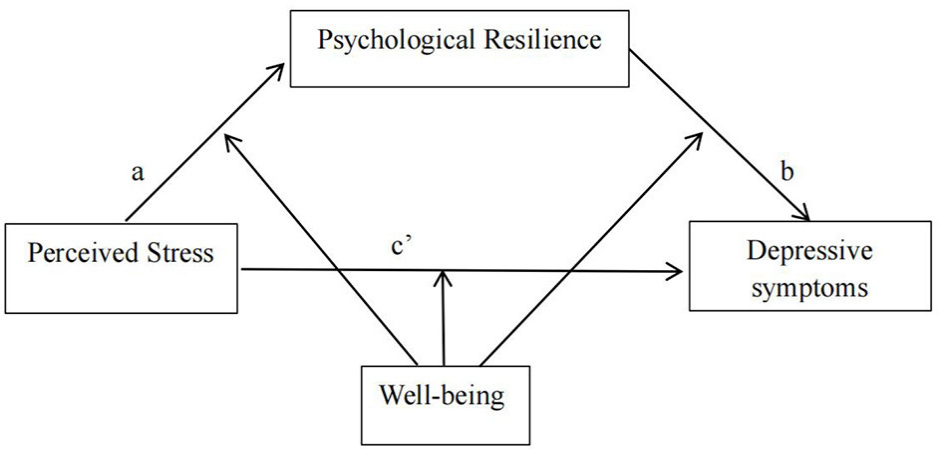
A schematic representation of wellbeing serving as a moderator within the mediation framework (Andrew Hayes's Moderation-Mediation Model 59).

Statistical analysis was performed utilizing SPSS 27.0 software. Descriptive analyses, independent *t*-tests, and one-way analysis of variance (ANOVA) were conducted to describe sociodemographic characteristics and compare the distribution of depressive symptoms, respectively. Pearson correlation analysis was employed to examine the relationships among psychological resilience, wellbeing, perceived stress, and depressive symptoms. The Harman single-factor test was utilized to assess common method bias. The mediation and moderated mediation models were analyzed using the PROCESS macro for SPSS (34). Bias-corrected 95% confidence intervals (CIs) were computed based on 5,000 bootstrap resamples. Initially, Model 4 (refer to Figure 1) was employed to explore whether the association between perceived stress and depressive symptoms was mediated by psychological resilience. If the 95% confidence interval (CI) for the indirect effect (path a ^*^ b) did not include 0, this signified a significant mediating effect. Subsequently, Model 59 was employed to assess the moderated mediation effect, specifically whether wellbeing moderated both the direct and indirect effects of perceived stress on depressive symptoms (see Figure 2). Similarly if the 95% confidence interval (CI) for the interaction term did not include 0, a significant moderated mediation effect was established. Additionally, all models accounted for covariates, including age, sex, family location, family type, single-child status, and annual household income, and all study variables were standardized. The significance level was set at α = 0.05.

## 3 Results

### 3.1 Common method deviation test

The Harman single-factor method was employed to test for common method bias, revealing 15 common factors. The first common factor explained 19.73% of the variance, which fell below the critical threshold of 40%. Hence, there was no significant common method bias present in this study (35).

### 3.2 Demographic data

The 299 participants included 98 males (32.8%) and 201 females (67.2%), aged between 20 and 23 years (mean age 21.51 ± 1.01). The sociodemographic characteristics and the distribution of depressive symptoms are shown in Table 1.

**Table 1:** Sociodemographic characteristics and the distribution of depressive symptoms (*N* = 299).

**Variables**		***N* (%)**	**Depressive symptoms (M ±SD)**	***F*/*t***	***P***
Gender	Males	98 (32.8)	9.41 ± 5.00	−1.35	0.179
	Females	201 (67.2)	10.27 ± 5.68		
Family location	City	119 (39.8)	9.91 ± 5.86		
	Village	180 (60.2)	10.05 ± 5.22	−0.215	0.830
Family type	Single parent family	23 (7.7)	9.52 ± 5.29		
	Two-parent family	267 (89.3)	10.01 ± 5.51	0.178	0.837
	Reorganized family	9 (3)	10.78 ± 5.26		
Only child	Yes	68 (22.7)	9.79 ± 5.78		
	No	231 (77.3)	10.05 ± 5.39	−0.328	0.743
Annual household income (yuan/year)	< 5,000	22 (7.4)	10.59 ± 4.49		
	5,000–20,000	73 (24.4)	10.10 ± 5.61		
	20,000–50,000	70 (23.4)	9.80 ± 5.30	0.73	0.572
	50,000–100,000	79 (26.4)	10.57 ± 5.56		
	> 100,000	55 (18.4)	9.04 ± 5.78		

Analysis revealed that the prevalence of moderate to severe depressive symptoms among undergraduate nursing interns in the final stages of their internships was 49.16% (PHQ-9 ≥ 10). Independent *t*-tests and ANOVA revealed no significant differences in depressive symptoms among undergraduate nursing interns across varying sociodemographic characteristics.

### 3.2 Descriptive statistics and correlation coefficient between variables

The findings revealed that undergraduate nursing interns exhibit average levels of wellbeing, psychological resilience, and depressive symptoms, alongside high levels of stress. Correlation analysis of the primary variables showed that wellbeing was positively associated with psychological resilience (*r* = 0.306, *p* < 0.01), yet negatively associated with perceived stress (*r* = −0.214, *p* < 0.01) and depressive symptoms (*r* = −0.615, *p* < 0.01). Psychological resilience showed negative correlations with perceived stress (*r* = −0.441, *p* < 0.01) and depressive symptoms (*r* = −0.431, *p* < 0.01), whereas perceived stress exhibited a positive correlation with depressive symptoms (*r* = 0.434, *p* < 0.01). Scores and correlation coefficients for each variable are detailed in Table 2.

**Table 2:** Descriptive statistics and correlation coefficient between variables (*N* = 299).

	**Variable score**	**Wellbeing**	**Perceived stress**	**Psychological resilience**	**Depressive symptoms**
Wellbeing	93.19 ± 18.56	1			
Perceived stress	30.09 ± 6.50	−0.214^**^	1		
Psychological resilience	23.26 ± 5.80	0.306^**^	−0.441^**^	1	
Depressive symptoms	9.99 ± 5.48	−0.615^**^	0.434^**^	−0.431^**^	1

^**^Indicates *P* < 0.01.

### 3.3 Mediation analyses

The SPSS macro program Process, developed by Hayes, was employed with model 4 selected. Depressive symptoms among undergraduate nursing interns were designated as the dependent variable, perceived stress as the independent variable, and psychological resilience as the mediating variable.

As presented in Table 3, the mediation analyses revealed that the total effect (path c) of perceived stress on depressive symptoms was statistically significant [B = 0.358, 95% CI (0.272, 0.444)]. The statistically significant coefficients for path a [B = −0.391, 95% CI (−0.482, −0.301)] and path b [B = −0.284, 95% CI (−0.387, −0.180)] denote negative associations between perceived stress and psychological resilience, as well as between psychological resilience and depressive symptoms. Furthermore, the point estimate of the indirect effect (path a ^*^ b) of perceived stress on depressive symptoms via psychological resilience was 0.111 [SE = 0.025, 95% CI (0.065, 0.162)], accounting for 31.01% of the total effect, demonstrating that the indirect effect was statistically significant. Additionally, the direct effect of perceived stress on depressive symptoms [path c' = 0.247, 95% CI (0.155, 0.338)] was significant, suggesting that psychological resilience partially mediated the association between perceived stress and depressive symptoms.

**Table 3:** Mediation analysis (*N* = 299).

**Variable**	**Path c**		**Paths c' and b**		**Path a**		**Path a** ^*^ **b**			
	**B**	**SE**	**B**	**SE**	**B**	**SE**	**B**	**SE**	**LLCI**	**ULCI**
Perceived stress	0.358^***^	0.044	0.247^***^	0.047	–	–	0.111^***^	0.025	0.065	0.162
Psychological resilience	–	–	−0.284^***^	0.053	−0.391^***^	0.046				
$R_{\text{adj}}^{2}$	0.189		0.262		0.196					
*F*	34.593		34.922		36.082					

Controlling for age, gender, family location, family type, only child, and annual household income.

^***^P < 0.001.

### 3.4 Moderated mediation analyses

Table 4 presents the results of the moderated mediation analysis. In line with the hypothesis, wellbeing may act as a moderator in the relationship between perceived stress and depressive symptoms, both in terms of the direct effect (perceived stress → depressive symptoms) and the indirect effect (path a: perceived stress → psychological resilience; path b: psychological resilience → depressive symptoms).

**Table 4:** Moderated mediation analysis (*n* = 299).

**Variable**	**B**	**SE**	***t***	**LLCI**	**ULI**
**Outcome: psychological resilience**					
Perceived stress	−0.351	0.046	−7.658^***^	−0.441	−0.261
Wellbeing	0.073	0.016	4.449^***^	0.041	0.105
Perceived stress^*^wellbeing	−0.001	0.002	−0.284	−0.005	0.004
**Outcome: depressive symptoms**					
Perceived stress	0.202	0.038	5.282^***^	0.127	0.277
Psychological resilience	−0.165	0.044	−3.705^***^	−0.252	−0.077
Wellbeing	−0.159	0.013	−12.184^***^	−0.185	−0.134
Psychological resilience^*^wellbeing	0.007	0.002	3.433^***^	0.003	0.011
Perceived stress^*^wellbeing	0.003	0.002	1.263	−0.001	0.006

Controlling for age, gender, family location, family type, only child, and annual household income.

^***^P < 0.001.

**Figure 3:**
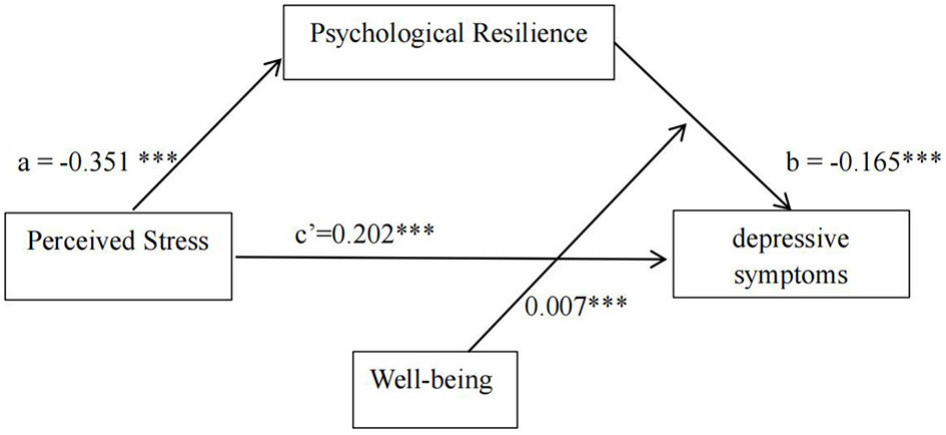
The final moderated mediation model (***P < 0.001).

However, wellbeing did not exert a moderating effect on the direct pathway [perceived stress ^*^ wellbeing: B = 0.003, 95% CI (−0.001, 0.006)] or the anterior pathway of the mediation model [perceived stress ^*^ wellbeing: B = −0.001, 95% CI (−0.005, 0.004)]. The results of the moderated mediation analysis revealed that wellbeing significantly moderated the posterior pathway of the mediation effects [psychological resilience ^*^ wellbeing: B = 0.007, 95% CI (0.003, 0.011)]. The results confirmed the hypothesis that wellbeing played a moderating role in psychological resilience and depressive symptoms. The final moderated mediation model is displayed in Figure 3.

**Figure 4:**
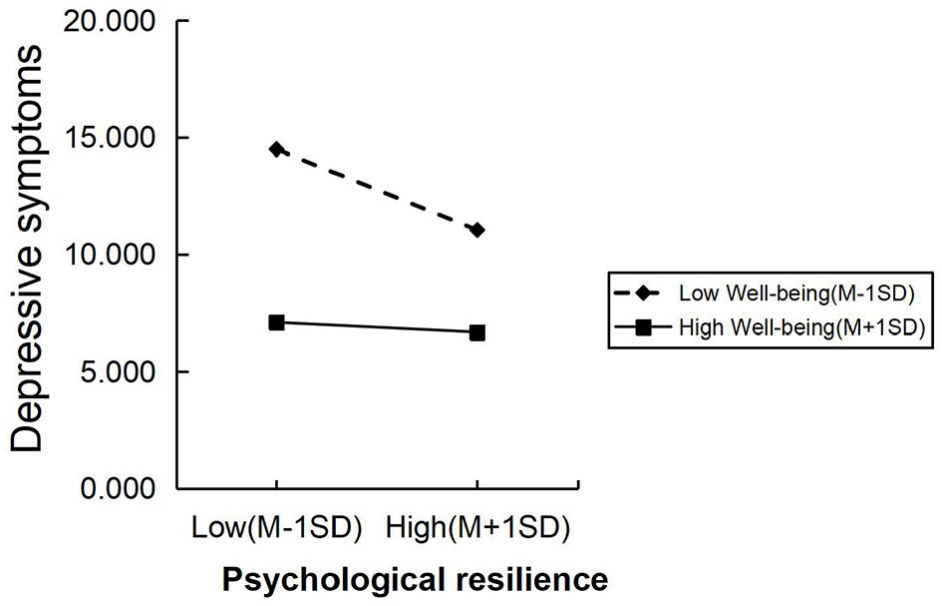
The simple slope test of the moderating effect of wellbeing on the relationship between psychological resilience and depressive symptoms.

To investigate the moderation effect, wellbeing was categorized into high and low groups using M ± 1 SD (Table 5). The simple slope test revealed that, compared to undergraduate nursing interns in the high wellbeing group, the impact of psychological resilience on depressive symptoms was significantly greater in the low wellbeing group (Figure 4).

**Table 5:** Conditional indirect effects of psychological resilience on depressive symptoms at values of wellbeing.

	**B**	**SE**	**LLCI**	**ULCI**
Low wellbeing	−0.294	0.061	−0.413	−0.174
Moderate wellbeing	−0.165	0.044	−0.252	−0.077
High wellbeing	−0.036	0.056	−0.146	0.074

## 4 Discussion

This cross-sectional survey aimed to assess psychological symptoms (perceived stress and depressive symptoms), psychological resilience, and wellbeing levels among undergraduate nursing interns during the mid and later stages of their internship. To date, this study is the first to investigate the moderating and mediating mechanisms of psychological resilience and wellbeing in the relationship between stress and depression, with psychological resilience serving as the mediating variable and wellbeing as the moderating variable.

Study findings revealed that undergraduate nursing students in the latter stages of their internships experienced heightened levels of perceived stress and depression, alongside moderate levels of wellbeing and psychological resilience. Consistent with earlier research (36, 37).

Central to the research findings is the examination of psychological resilience and wellbeing as intervening variables in the relationship between perceived stress and depressive symptoms within the proposed model. The study findings revealed that psychological resilience significantly mediates the relationship between perceived stress and depressive symptoms, while wellbeing plays a significant moderating role in the association between psychological resilience and depressive symptoms. Additionally, the findings provided new insights into the significant moderating role of wellbeing. Specifically, the moderating effect of wellbeing on the relationship between psychological resilience and depressive symptoms was significant only at lower levels of wellbeing.

In the mediation effect analysis, direct path analysis (Perceived Stress → Depressive symptoms) revealed that perceived stress directly predicts depressive symptoms positively, suggesting that higher perceived stress leads to a greater propensity for depression. These findings were consistent with previous studies (16, 38–40), which had shown that perceived stress increased the risk of depression in college students. This conclusion was supported by neural mechanism studies, indicating that high perceived stress was associated with structural, functional, and connectivity changes in certain brain regions. These regions primarily include the prefrontal cortex, hippocampus, and amygdala, all part of the limbic system (41–43). A stable and significant positive association was found between abnormalities in these brain areas and the severity of depressive symptoms (44). Nursing students were encouraged to employ problem-focused (focused on problem-solving), emotion-focused (focused on managing emotions), and dysfunctional (focused on venting emotions) stress coping strategies to mitigate stress (12).

Psychological resilience partially mediated the relationship between perceived stress and depressive symptoms. It indicates that perceived stress can impact depressive symptoms in undergraduate nursing interns through psychological resilience. Individuals with high psychological resilience scores were likely to mobilize their resilience resources to manage stress and mitigate adverse consequences. Therefore, enhancing an individual's psychological resilience could lessen the negative effects of stress and consequently reduce the incidence of depression. The findings align with theories of resilience (45) and prior studies (16, 38, 46).

The moderation analysis revealed a significant dynamic effect of wellbeing on the relationship between psychological resilience and depressive symptoms. Compared to undergraduate nursing interns with high wellbeing, the impact of psychological resilience in alleviating depressive symptoms was more pronounced among those with low wellbeing. Specifically, when wellbeing was below a certain threshold (which varied by sample), the impact of psychological resilience on alleviating depressive symptoms became more pronounced with increasing wellbeing scores. However, once wellbeing reached a critical level, the moderating effect became non-significant. This effect may be interpreted in two ways. First, individuals with low wellbeing might struggle to effectively utilize psychological resilience when under stress. Second, high wellbeing is associated with lower levels of depression (46), and the enhancing interaction between wellbeing and psychological resilience means that individuals with high wellbeing experience limited additional benefits from psychological resilience. Therefore, for undergraduate nursing interns with high wellbeing, if depressive symptoms arise, seeking additional psychological resources may be necessary for relief.

## 5 Limitations and prospects

The study is not free from limitations. Due to its cross-sectional observational design, the causal relationships among these factors were not conclusively established; rather, the focus was on exploring the underlying mechanisms.

Future research should include longitudinal studies to further validate and substantiate these findings.

## 6 Theoretical and practical implications

The value of the present study was to verify the resilience theory and the dynamic role of wellbeing in alleviating perceived stress and depressive symptoms among undergraduate nursing interns in the later stages of their internships. First, this study found that psychological resilience mediated the relationship between perceived stress and depressive symptoms, supporting the appraisal mechanism of resilience in protecting individual mental health. Specifically, psychological resilience can lessen the impact of perceived stress on depressive symptoms among undergraduate nursing interns. Universities should implement courses on psychological resilience training, emphasizing cognitive and emotional regulation, behavioral training, and mental regulation. Hospitals should create social media platforms during internships to offer emotional support channels for nursing interns. Second, the study demonstrated that wellbeing moderates the relationship between psychological resilience and depressive symptoms. Notably, it has been observed that the moderating effect of wellbeing is significant solely at low levels of wellbeing. Therefore, interventions aimed at improving wellbeing could enhance psychological resilience, thereby further reducing perceived stress and depressive symptoms. Nursing educators and administrators should organize activities to enhance the wellbeing of undergraduate nursing interns with low wellbeing. Undergraduate nursing interns with high wellbeing should maintain their current status to keep perceived stress and depressive symptoms low, thereby safeguarding their mental health.

## 7 Conclusion

This study investigated the mediating role of psychological resilience and the moderating effect of wellbeing on the relationship between perceived stress and depressive symptoms. The findings indicated a significant association between perceived stress and depressive symptoms, with psychological resilience serving as a mediator in this relationship. Additionally, wellbeing moderated the latter portion of the mediated pathway, specifically the link between psychological resilience and depressive symptoms. Specifically, the alleviating effect of wellbeing was more pronounced at lower levels of wellbeing.

## Data Availability

The raw data supporting the conclusions of this article will be made available by the authors, without undue reservation.
